# A novel bystander effect in tamoxifen treatment: PPIB derived from ER+ cells attenuates ER− cells via endoplasmic reticulum stress-induced apoptosis

**DOI:** 10.1038/s41419-024-06539-3

**Published:** 2024-02-15

**Authors:** Tinglin Yang, Wenhui Li, Jun Zhou, Ming Xu, Ziwei Huang, Jie Ming, Tao Huang

**Affiliations:** Department of Breast and Thyroid Surgery, Union Hospital, Tongji Medical College, Huazhong University of Science and Technology, Wuhan, 430022 China

**Keywords:** Breast cancer, Cancer therapy

## Abstract

Tamoxifen (TAM) is the frontline therapy for estrogen receptor-positive (ER+) breast cancer in premenopausal women that interrupts ER signaling. As tumors with elevated heterogeneity, amounts of ER-negative (ER−) cells are present in ER+ breast cancer that cannot be directly killed by TAM. Despite complete remissions have been achieved in clinical practice, the mechanism underlying the elimination of ER− cells during TAM treatment remains an open issue. Herein, we deciphered the elimination of ER− cells in TAM treatment from the perspective of the bystander effect. Markable reductions were observed in tumorigenesis of ER− breast cancer cells by applying both supernatants from TAM-treated ER+ cells and a transwell co-culture system, validating the presence of a TAM-induced bystander effect. The major antitumor protein derived from ER+ cells, peptidyl-prolyl cis-trans isomerase B (PPIB), is the mediator of the TAM-induced bystander effect identified by quantitative proteomics. The attenuation of ER− cells was attributed to activated BiP/eIF2α/CHOP axis and promoted endoplasmic reticulum stress (ERS)-induced apoptosis, which can also be triggered by PPIB independently. Altogether, our study revealed a novel TAM-induced bystander effect in TAM treatment of ER+ breast cancer, raising the possibility of developing PPIB as a synergistic antitumor agent or even substitute endocrine therapy.

## Introduction

As the most common malignancy worldwide, breast cancer is classified into different subtypes according to the expression levels of hormone receptors (HRs) estrogen receptor (ER) and progesterone receptor (PR), and human epidermal growth factor receptor-2 (HER2) [1]. Estrogen receptor-positive (ER+) breast cancer is the predominant subtype among breast cancers, accounting for ~70% of cases. The mainstream treatment for ER+ breast cancer is endocrine therapy, which aims to disrupt ER signaling [2]. As one of the most widely administered endocrine therapy agents, tamoxifen (TAM) is well recognized as a frontline treatment for premenopausal women patients, leading to a 40% reduction in recurrences and a 30% reduction in deaths [3, 4]. Owing to the heterogeneous nature of ER+ breast cancer, the ER+ tumor mass demonstrated by ≥1% ER-positive stained tumor cells in immunohistochemistry (IHC) tests actually contains various amounts of ER-negative (ER−) cells [5]. Since ER is the therapeutic target of TAM, only ER+ cancer cells can be directly killed in TAM treatment according to its antagonizing effect of ER [6]. Despite TAM seeming to be unfacilitated to directly kill ER− cells, complete remissions can be achieved by TAM treatment in clinical practice, including neoadjuvant settings [7–9]. Therefore, the mechanism underlying the elimination of ER− cells during TAM treatment remains an open issue worth exploring.

A similar situation in cancer treatment was observed and defined as the bystander effect, which refers to the phenomenon that therapy-targeted tumor cells can secret factors to affect untargeted neighboring cells, resulting in attenuated cell proliferation, senescence, or even cell death [10]. The bystander effect has been researched in radiation therapy and treatment with antibody-drug conjugates [11, 12]. However, TAM treatment has not been reported to show connections with the bystander effect.

In this study, we deciphered the mechanism underlying the elimination of ER− cells in TAM treatment in breast cancer from the perspective of the bystander effect. We identified a markable reduction in tumorigenesis of ER− breast cancer cells by applying both supernatants from TAM-treated ER+ cells and a transwell co-culture system, validating the presence of a TAM-induced bystander effect. The intratumoral injection model in nude mice further consolidated our observation of the bystander effect in vivo. To further illustrate the mechanism underlying the TAM-induced bystander effect, we exploited proteomic analysis, identifying peptidyl-prolyl cis-trans isomerase B (PPIB) as the pivotal mediator of the TAM-induced bystander effect. Moreover, RNA sequencing in the ER− cells showed significantly increased expression of growth differentiation factor 15 (GDF15), a protein that can be upregulated when suffering endoplasmic reticulum stress (ERS) [13]. ERS is a response to the overload of misfolded protein in the endoplasmic reticulum lumen, and mild ERS that promotes proliferation can be detected in breast cancer development. However, prolonged, severe, and overwhelmed ERS is irreversible and expedites cell death, which is deemed a potential target for antitumor therapy [14]. Our results revealed the activation of the BiP/eIF2α/CHOP axis in the ER− cells, which is a typical axis indicating the presence of a sustained ERS that eventually led to apoptosis. The enhancement of ERS and the upregulation of GDF15 can also be induced by independently treating ER− cells with PPIB recombinant protein, confirming the predominant role PPIB played in the TAM-induced bystander effect.

Taken together, our study identified a novel bystander effect in TAM treatment, giving new insights into comprehending the eradication of ER− cells. The material basis of the TAM-induced bystander effect, PPIB, was also proved, indicating a potential antitumor therapeutic strategy.

## Results

### Conditioned medium from TAM-treated ER+ breast cancer cells selectively attenuates tumorigenesis of ER− cells

**Fig. 1:**
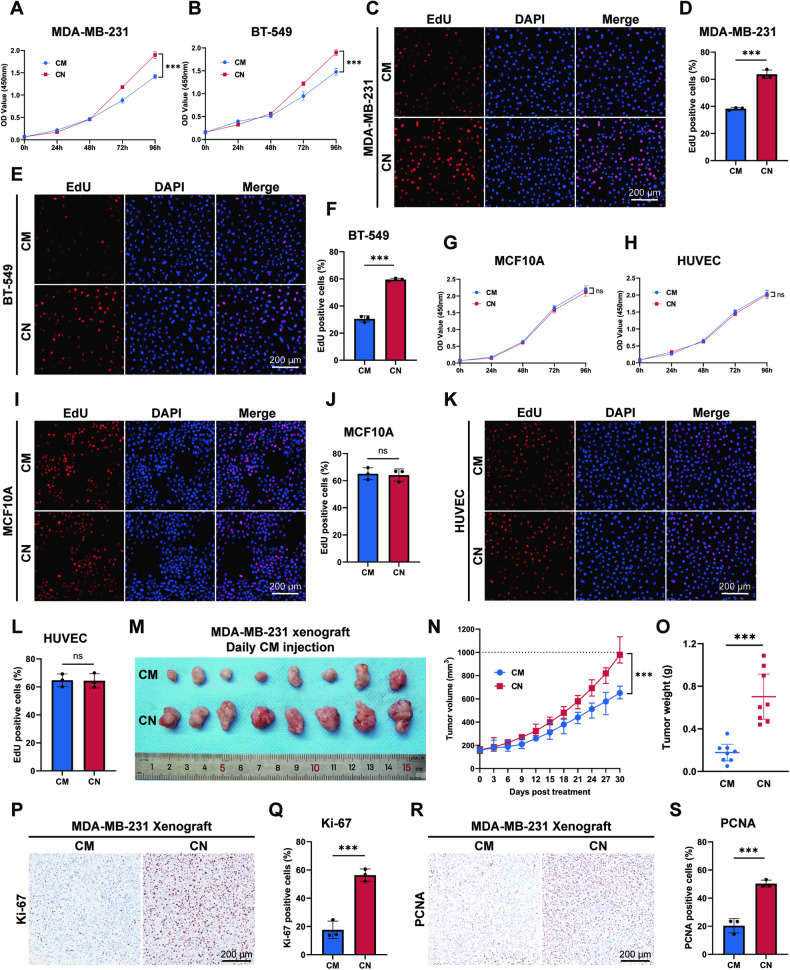
Conditioned medium from TAM-treated MCF7 cells selectively attenuates tumorigenesis of ER− cells. CM conditioned medium, CN ctrl medium. ***p value < 0.001. **A**, **B** ER− breast cancer cell lines MDA-MB-231 and BT-549 were cultured with CM or CN generated from MCF7 cells. Inhibited viability of MDA-MB-231 and BT-549 cells cultured with CM was detected by CCK-8 assays. *n* = 5. **C**, **D** The repressed proliferation of MDA-MB-231 cells cultured with CM was assessed by EdU assays. *n* = 3. **E**, **F** The repressed proliferation of BT-549 cells cultured with CM was assessed by EdU assays. *n* = 3. **G**, **H** Human normal breast epithelial cell line MCF10A and the human umbilical vein endothelial cell (HUVEC) were cultured with CM or CN. CM showed no suppression of viability of MCF10A and HUVEC cells in CCK-8 assays. *n* = 5. **I**, **J** CM showed no repression to the proliferation of MCF10A cells in EdU assays. *n* = 3. **K**, **L** CM showed no repression to the proliferation of HUVEC cells in EdU assays. *n* = 3. **M**–**O** Serum-free CM or CN was intratumorally injected daily into MDA-MB-231 xenografts. Reduction in tumor volume and weight was measured in the CM group. *n* = 8. **P**, **Q** Decreased Ki-67 expressions in CM-injected MDA-MB-231 xenografts. **R**, **S** Decreased PCNA expressions in CM-injected MDA-MB-231 xenografts.

To preliminarily evaluate if there exists a bystander effect in TAM treatment, we exposed ER− breast cancer cells to medium conditioned by TAM-treated ER+ cells. The conditioned medium (CM) was prepared by treating the ER+ breast cancer cell line MCF7 with TAM for 24 h, followed by rinsing with PBS. After culturing for additional 24 h, MCF7 CM was collected. For the control medium (CN), supernatant from MCF7 cells treated with equal concentrations of dimethyl sulfoxide (DMSO) was collected. The ER− cell lines MDA-MB-231 and BT-549 were then cultured with CM or CN to determine the presence of antitumor factors in CM. In comparison to CN, CM demonstrated significant efficacy in inhibiting cell viabilities of MDA-MB-231 and BT-549 cells, as detected in CCK-8 assays (Fig. 1A, B). After cultivating for 72 h, ER− cells were harvested to undergo subsequent experiments. Consistent with results in CCK-8 assays, both MDA-MB-231 and BT-549 cells exhibited compromised proliferation ability in EdU assays (Fig. 1C–F). Notable declined colony-forming capacity (Fig. S1A–C), percentage of Ki-67 positive cells (Fig. S1D–F), and PCNA expressions (Fig. S1G) further validated the reduced proliferation capacity in CM-cultured MDA-MB-231 and BT-549 cells. Impaired migration ability was also demonstrated in transwell assays (Fig. S1H–J) and wound healing assays (Fig. S1K–P), as well as increased E-cadherin and decreased N-cadherin expressions in CM-cultured MDA-MB-231 and BT-549 cells (Fig. S1Q). These findings collectively suggest that CM attenuates tumorigenesis in ER− cells.

CM from another ER+ cell line, T47D, was generated and found to inhibit the growth of BT-549 cells (Fig. S1R), thereby validating the existence of the TAM-induced bystander effect. In contrast, CM from MCF7 cells did not exhibit any inhibitory effects on the normal cell lines, including MCF10A or HUVEC, as evidenced by CCK-8-based cell viability (Fig. 1G, H) and EdU-based cell proliferation (Fig. 1I–L). Thus, the selective nature of bystander toxicity to ER− cells is demonstrated.

We further established subcutaneous xenograft tumor models with MDA-MB-231 cells in nude mice to mimic the bystander effect in vivo. Daily intratumoral injections of freshly generated, serum-free MCF7 CM or CN were administered, resulting in attenuated tumor growth that reduced tumor volumes and weights detected in CM-injected tumors (Fig. 1M–O). Decreased Ki-67 expressions (Fig. 1P, Q) and PCNA expressions (Fig. 1R, S) in CM-injected tumor tissues also suggested attenuated tumor proliferation. Therefore, our results support the identification of TAM-induced bystander effect both in vitro and in vivo, indicating that CM of ER+ cells contains specific factor(s) that selectively attenuate ER− breast cancer cells.

### ER− cells are suppressed by TAM in co-culture models with ER+ cells

**Fig. 2:**
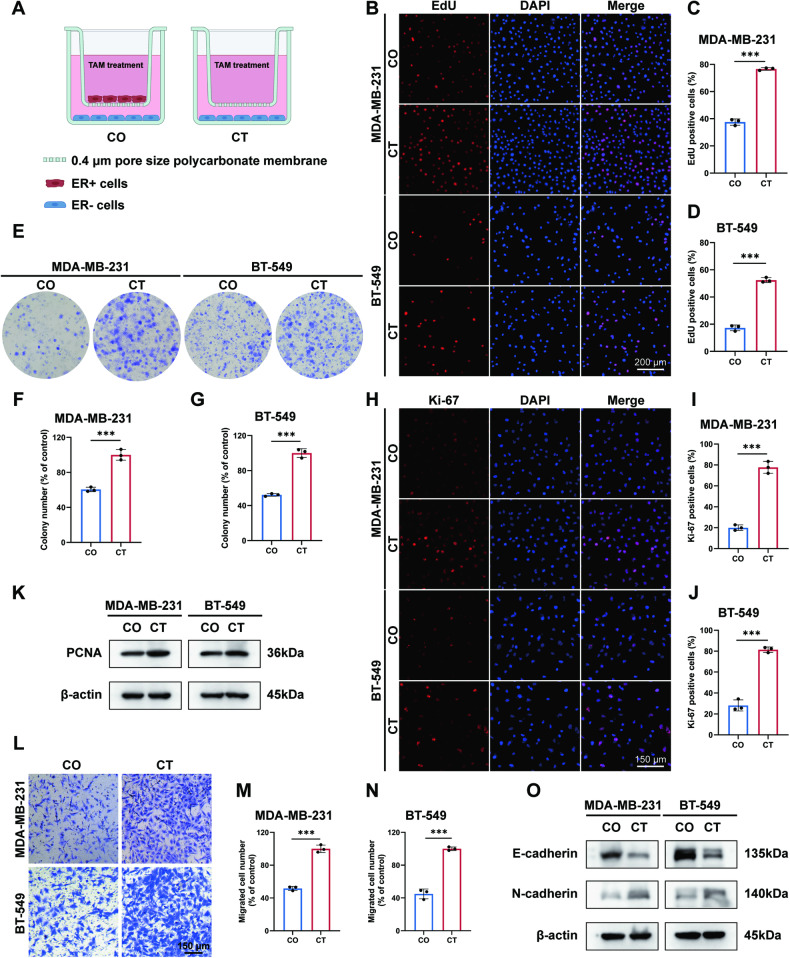
ER− cells are suppressed by TAM when co-culturing with MCF7 cells. CO co-culture group, CT control group. ***p value < 0.001. *n* = 3. **A** The scheme of the transwell co-culture system. **B**–**D** Diminished proliferation capacity of MDA-MB-231 and BT-549 cells in the condition of co-culturing with MCF7 cells. **E**–**G** Declined colony-forming ability of MDA-MB-231 and BT-549 cells co-cultured with MCF7 cells. **H**–**J** Reduced percentage of Ki-67 positive MDA-MB-231 and BT-549 cells co-cultured with MCF7 cells. **K** PCNA expressions were decreased in MDA-MB-231 and BT-549 cells co-cultured with MCF7 cells. **L**–**N** Transwell-based migration was suppressed in MDA-MB-231 and BT-549 cells co-cultured with MCF7 cells. **O** MDA-MB-231 and BT-549 cells exhibited increased E-cadherin levels and decreased N-cadherin levels when co-culturing with MCF7 cells.

As an approach mimicking the in vivo environment, CM provided the opportunity to observe the presence of antitumoral soluble factors in TAM-treated MCF7 supernatant. However, it was unclear whether the feedback from ER− cells would neutralize the antitumor effect. A transwell co-culture system was established to exclude the interruption of the TAM-induced bystander effect by crosstalk between cells. In the co-culture group (CO), MCF7 cells were seeded on the 0.4-μm pore size polycarbonate membrane in the upper chamber, while ER− cells MDA-MB-231 or BT-549 were seeded in the lower chamber. In the control group (CT), only ER− cells were seeded in the lower chamber. Following adhesion, the cells were exposed to TAM at an equivalent concentration for 72 h (Fig. 2A). Although the cells were unable to traverse the membrane of the chamber, soluble factors, and small molecules were able to permeate and interact. Coculturing MDA-MB-231 and BT-549 cells with MCF7 cells led to diminished proliferation capacity (Fig. 2B–D) and declined colony-forming capacity (Fig. 2E–G). Consistently, reductions in the percentage of Ki-67 positive cells (Fig. 2H–J) and PCNA expressions (Fig. 2K) were detected in co-cultured cells. Impaired transwell-based migration (Fig. 2L–N) and hindered scratch-based migration (Fig. S2A, B) were also observed in co-cultured MDA-MB-231 and BT-549 cells, accompanied by the upregulation of E-cadherin and downregulation of N-cadherin expressions (Fig. 2O). The co-culture system further validates the presence of the TAM-induced bystander effect, which is not neutralized by the interplay between ER− and ER+ cells.

### Proteomic analysis revealed a markable increase in PPIB in CM

Label-free quantitative proteomics was utilized to identify the pivotal antitumor protein. Serum-free CM and CN of TAM-treated or DMSO-treated MCF7 cells were collected, followed by Coomassie Brilliant Blue staining of proteins in supernatants after SDS-PAGE. The CM group exhibited higher numbers of protein bands and greater protein concentrations (Fig. S3A). Quantitative mass spectrometry analysis was performed on the CM and CN samples (*n* = 3 for each group), and the peptide coverage of proteins and correlations of samples were analyzed to ensure data quality (Fig. S3B, C).

**Fig. 3:**
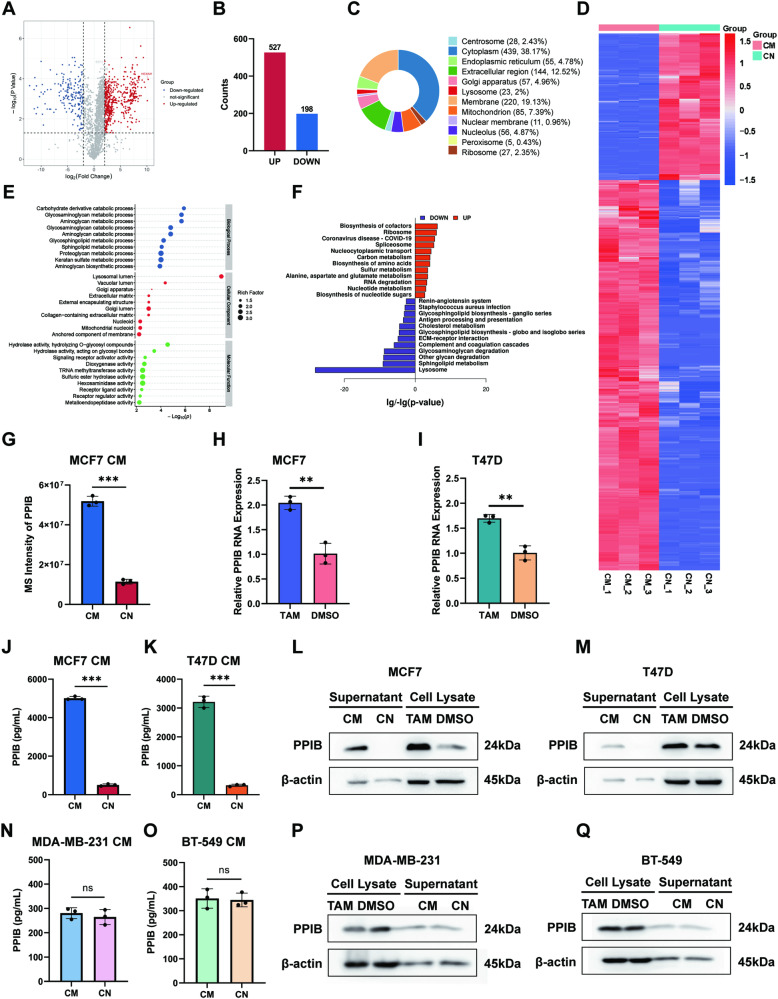
PPIB is markedly increased in TAM-treated MCF7 CM. CM conditioned medium, CN control medium. ***p value < 0.001. *n* = 3. **A** MCF7 cells were treated with TAM or DMSO to generate serum-free CM or CN, which subsequently underwent label-free quantitative proteomic analysis. Volcano plot of DEPs in MCF7 CM compared to the CN was plotted. **B** Numbers of upregulated and downregulated DEPs in CM. **C** Subcellular locations of DEPs. **D** Heat map visualizing the clustering patterns of DEPs in the CM group and CN group. **E** GO analysis of the top enriched terms of DEPs. **F** KEGG enrichment of regulated pathways. **G** The upregulated MS intensity of PPIB in CM. **H**, **I** Increased PPIB RNA levels in MCF7 cells and T47D cells treated with TAM. **J**, **K** Increased PPIB levels in CM of MCF7 and PPIB detected by ELISA assays. **L**, **M** Increased PPIB levels in both supernatant and cell lysate of MCF7 and T47D cells treated with TAM. **N**, **O** Extracellular PPIB levels in MDA-MB-231 and BT-549 cells showed no statistical difference in response to TAM in ELISA assays. **P**, **Q** Intracellular and extracellular PPIB levels in TAM-treated MDA-MB-231 and BT-549 cells were not upregulated.

A total of 2357 proteins were identified through mass spectrometry analysis. Among these, the CM group exhibited 227 unique proteins, while the CN group had 31 unique proteins. The remaining 2099 proteins were detected in both the CM and CN groups (Fig. S3D). An amount of 725 differentially expressed proteins (DEPs) were screened with a threshold of *p* value < 0.05, |log2FC| >2, thereinto, 527 upregulated DEPs and 198 downregulated DEPs (Fig. 3A, B). The subcellular localizations of DEPs were annotated and investigated by analyzing the cellular component of the Gene Ontology (GO) knowledgebase, which revealed that DEPs were present in a variety of subcellular locations (Fig. 3C). Heat maps were generated to visualize the clustering patterns of DEPs in the CM group and CN group, illustrating stable inter-group differences and high degrees of intra-group similarities (Fig. 3D). Additionally, enrichment analyses were conducted using GO and Kyoto Encyclopedia of Genes and Genomes (KEGG) databases. The top 10 enriched GO terms for biological processes, cellular components, and molecular functions were reported (Fig. 3E). The top 12 enriched upregulated and downregulated KEGG pathways of DEPs were annotated, among which metabolic pathways were significantly enriched (Fig. 3F). Focusing on antitumor potentials, possible secretory proteins, and protein abundance, we proposed 19 tumor-suppressive candidates. By validating intracellular mRNA expression levels of the 19 candidates in TAM-treated MCF7 cells, we considered PPIB as the major antitumor protein due to its stable mRNA upregulation, high fold change, and reliable protein abundance (Fig. S3E). The MS intensity of PPIB in the CM group was four times higher than that in the CN group in label-free quantification (Fig. 3G).

Next, we provided the upregulation of PPIB with experimental evidence. MCF7 cells and T47D cells were subjected to a 24-h treatment with TAM or DMSO, followed by the collection of cells after 24 h of drug elusion. The serum-free supernatants of TAM-treated or DMSO-treated cells were also generated. TAM treatment resulted in a significant increase in intracellular mRNA and protein expression levels of PPIB (Fig. 3H, I, L, M). To confirm the enrichment of PPIB in the supernatant, enzyme-linked immunosorbent assay (ELISA) and western blotting assays were conducted in supernatants from MCF7 cells and T47D cells. Notably, ELISA assays revealed 9-fold higher concentrations in both MCF7 CM and T47D CM (Fig. 3J–M). Considering the heterogeneous nature of ER+ breast cancer, we also tested if PPIB was upregulated in TAM-treated ER− cells. Both intracellular and extracellular PPIB levels in MDA-MB-231 and BT-549 cells exhibited no increase in response to TAM (Fig. 3N–Q), suggesting that PPIB is specifically derived from ER+ cells in TAM-induced bystander effect.

### PPIB is the major antitumor protein that attenuates ER− cells

**Fig. 4:**
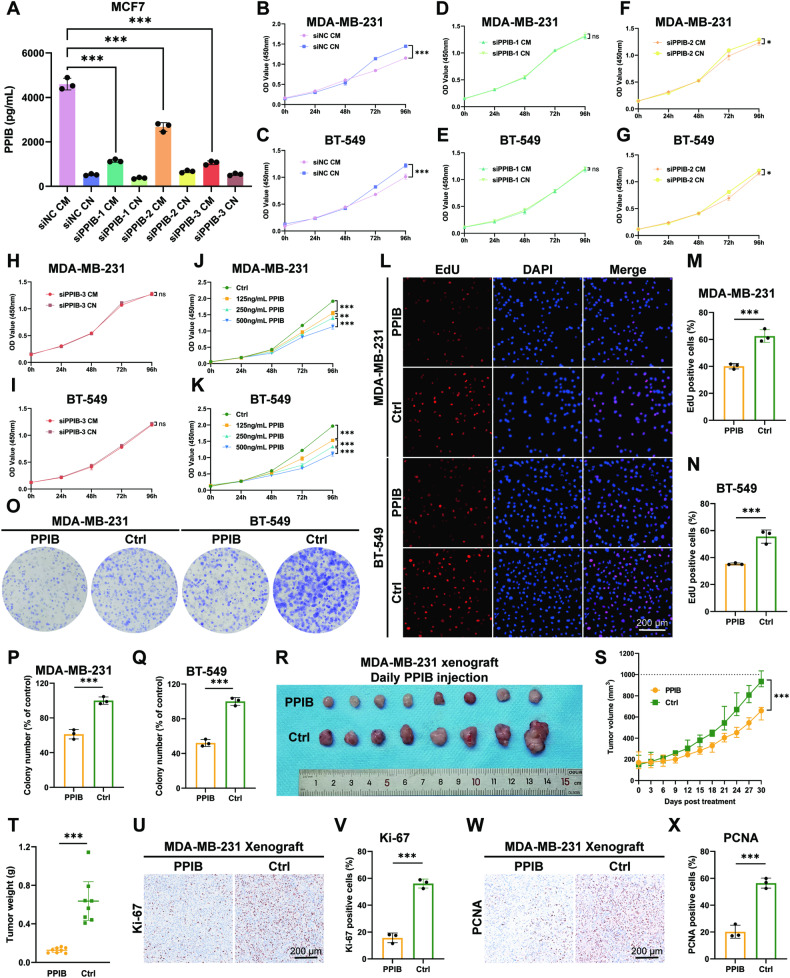
PPIB is the predominant antitumor protein in MCF7 CM. CM conditioned medium, CN control medium. *p value < 0.05, **p value < 0.01, ***p value < 0.001. **A** Decreased PPIB levels in CM by knocking down PPIB in MCF7 cells. *n* = 3. **B**, **C** CM from MCF7 siPPIB-NC cells repressed the viability of MDA-MB-231 and BT-549 cells. *n* = 5. **D**, **E** CM from MCF7 siPPIB-1 cells showed no repression to the viability of MDA-MB-231 and BT-549 cells. *n* = 5. **F**, **G** CM from MCF7 siPPIB-2 cells showed diminished repression to the viability of MDA-MB-231 and BT-549 cells. *n* = 5. **H**, **I** CM from MCF7 siPPIB-3 cells showed no repression to the viability of MDA-MB-231 and BT-549 cells. *n* = 5. **J**, **K** MDA-MB-231 and BT-549 cells were treated with PPIB at concentrations of 125, 250, and 500 ng/mL. Inhibition of cell viability increased with increasing concentrations. *n* = 5. **L**–**N** Attenuated proliferation was detected in MDA-MB-231 and BT-549 cells treated with 125 ng/mL PPIB. *n* = 3. **O**–**Q** Fewer colonies were formed in PPIB-treated MDA-MB-231 and BT-549 cells. *n* = 3. **R**–**T** PPIB was intratumorally injected daily into MDA-MB-231 xenografts. Reduction in tumor volume and weight was measured. *n* = 8. **U**, **V** Decreased Ki-67 expression was detected in PPIB-injected MDA-MB-231 xenografts. **W**, **X** Decreased PCNA expression was detected in PPIB-injected MDA-MB-231 xenografts.

MCF7 cells were transfected with control siRNA (siNC) or siRNA targeting PPIB (siPPIB). The successful knockdown of PPIB by siPPIB-1, -2, and -3 was confirmed using qPCR and western blotting (Fig. S4A, B). The knockdown of secreted PPIB was verified by conducting ELISA and western blotting in the CM or CN from MCF7 siNC and MCF7 siPPIB cells (Fig. 4A and Fig. S4C). The viability of MDA-MB-231 cells and BT-549 cells was repressed by MCF7 siNC CM (Fig. 4B, C), whereas MCF7 siPPIB-1 CM and MCF7 siPPIB-3 CM exhibited no antitumor effect (Fig. 4D, E, H, I). The MCF7 siPPIB-2 CM showed a mitigated antitumor effect in comparison to MCF7 siNC CM, aligning with the diminished knockdown efficacy of siPPIB-2 (Fig. 4F, G). The attenuation of ER− cells can be rescued by PPIB knockdown in MCF7, indicating the pivotal role of PPIB as an antitumor protein in MCF7 CM.

The application of PPIB recombinant protein at concentrations of 125, 250, and 500 ng/mL significantly suppressed cell viability in MDA-MB-231 cells and BT-549 cells, with a notable enhancement in suppression along with the increasing PPIB concentrations (Fig. 4J, K). The cell proliferation, colony formation ability, and migration ability of MDA-MB-231 cells and BT-549 cells were also attenuated by the administration of PPIB recombinant protein (Fig. 4L–Q and Fig. S4D–F). The decreased percentage of Ki-67 positive cells (Fig. S4G–J) and reduced PCNA expressions (Fig. S4I) were accordantly detected in PPIB-treated MDA-MB-231 cells and BT-549 cells. Furthermore, the intratumoral injection of PPIB recombinant protein led to the repression of MDA-MB-231 subcutaneous xenograft tumor growth in nude mice. This was evidenced by smaller tumor volumes and lighter tumor weights (Fig. 4R–T), as well as decreased expressions of Ki-67 (Fig. 4U, V) and PCNA (Fig. 4W, X), demonstrating the in vivo antitumor effect of PPIB. Therefore, the antitumor protein PPIB exhibits the ability to suppress tumorigenesis of ER− cells and plays a prominent role in the TAM-induced bystander effect.

### Stress-responsive protein GDF15 is enriched in ER− cells in TAM-induced bystander effect

**Fig. 5:**
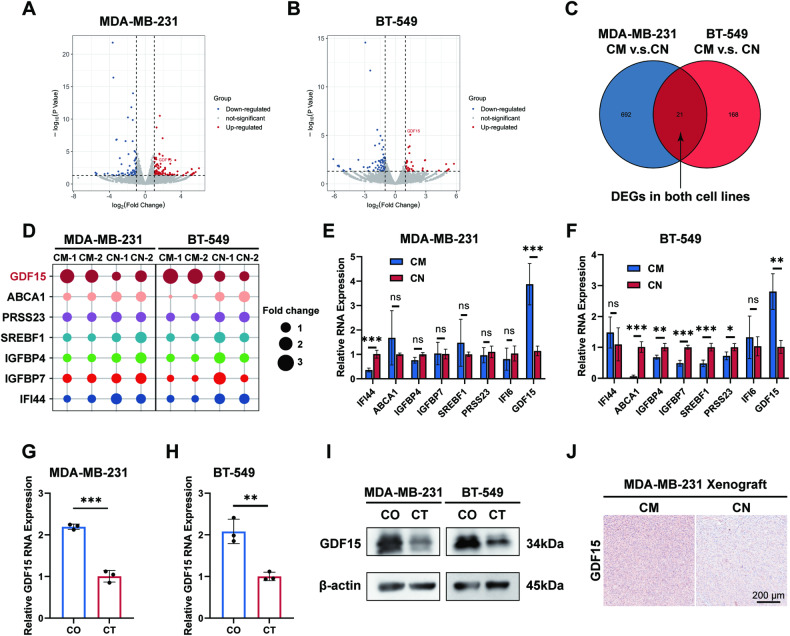
Stress-responsive protein GDF15 is upregulated in ER− cells in TAM-induced bystander effect. CM conditioned medium, CN control medium, CO co-culture group, CT control group. *p value < 0.05, **p value < 0.01, ***p value < 0.001. *n* = 3. **A**, **B** Volcano plots of DEGs in CM-cultured MDA-MB-231 cells and BT-549 cells compared to the CN-cultured cells. **C** Venn plot of the commonly regulated genes in both cell lines. **D** Bubble plot visualizing the seven commonly upregulated or downregulated genes (fold change). **E**, **F** Levels of DEGs verified by qPCR in MDA-MB-231 and BT-549 cells cultured with CM or CN. **G**, **H** Upregulated GDF15 RNA levels in MDA-MB-231 and BT-549 cells co-cultured with MCF7 cells. **I** Increased GDF15 expressions in MDA-MB-231 and BT-549 cells co-cultured with MCF7 cells. **J** Increased GDF15 expression in MDA-MB-231 xenografts intratumorally injected CM daily.

To analyze the mechanism underlying the attenuation of ER− cells, we conducted RNA sequencing in MDA-MB-231 cells and BT-549 cells cultured with CM or CN. Principal component analysis (PCA) was performed and depicted (Fig. S5A). The gene expression profiles of both cell lines were significantly altered while preserving cell-type specificity. A total of 713 differentially expressed genes (DEGs) were identified in MDA-MB-231 cells, and 189 DEGs were found in BT-549 cells (*p* value < 0.05) (Fig. 5A, B). The top enriched KEGG pathways in MDA-MB-231 and BT-549 were analyzed, along with the cluster analysis of DEGs within and between groups (Fig. S5B, D). In both cell lines, 21 genes were found to be significantly differentially expressed (Fig. 5C). The top seven DEGs across both cell lines were depicted, among which GDF15 denoted a pronounced and consistent change of mRNA expression in MDA-MB-231 and BT-549 cells (Fig. 5D). The top DEGs were also examined by qPCR assays conducted in CM-cultured MDA-MB-231 and BT-549 cells, confirming significant upregulated GDF15 expressions (Fig. 5E, F). GDF15 is a protein belonging to the TGFβ superfamily and is known to be induced by stress response [15]. Experimental evidence has shown that GDF15 expression in cancer cells can be upregulated by drugs, and upregulation of GDF15 presents in tumor-suppressive circumstances [16–18]. These results aligned with the increase of GDF15 in attenuated ER− cells in our study.

The upregulation of GDF15 expression at both mRNA level and protein level was approved by our results in MDA-MB-231 and BT-549 cells co-cultured with MCF7 (Fig. 5G–I). Besides, upregulated GDF15 was also verified by both RNA sequencing data (Fig. S5E, F) and western blotting (Fig. S5G) in CM- cultured ER− cells. In an in vivo setting, the IHC assay revealed increased expression of GDF15 in mice subcutaneous tumors that were intratumorally injected with CM daily (Fig. 5J).

### MCF7 CM kills ER− cells by activating ERS-induced apoptosis

Next, we sought to elucidate the mechanism of upregulated GDF15 in ER− cells. Previous work has shown that GDF15 is a stress-responsive protein that exhibits increased expression in response to diverse stress environments, including ERS [15]. The expression of C/EBP homologous protein (CHOP) can be enhanced in irreversible ERS and serves as a crucial transcription factor promoting the upregulation of GDF15 [19, 20].

**Fig. 6:**
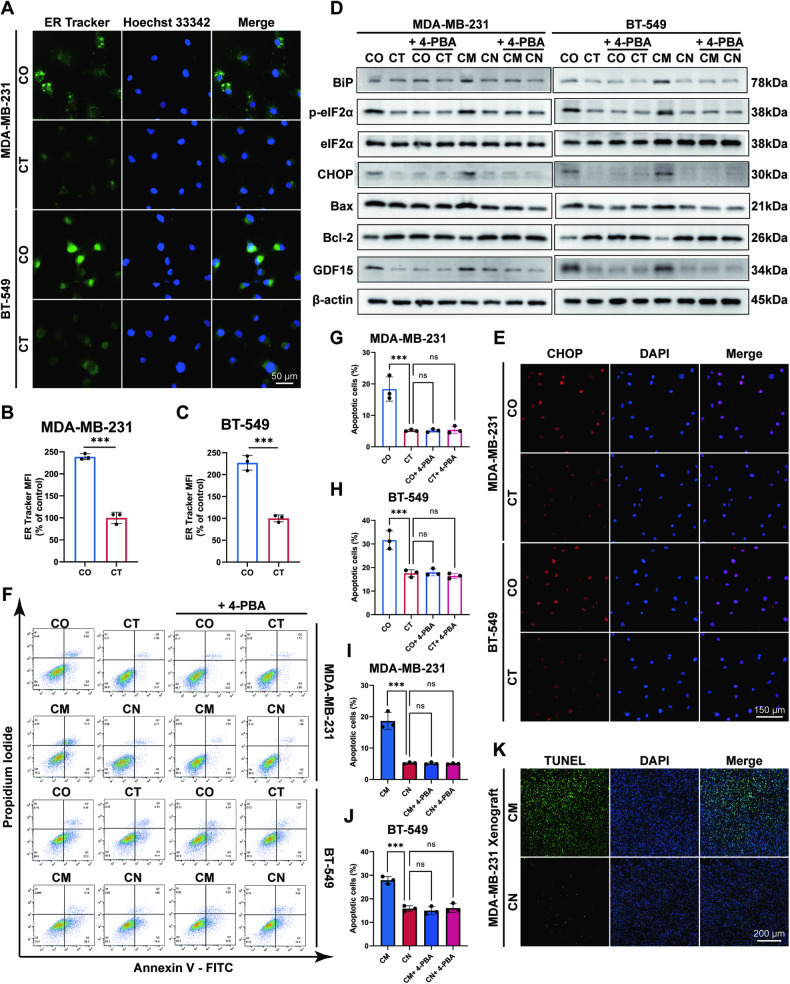
ERS-induced apoptosis is activated in ER− cells in bystander effect. CM conditioned medium, CN control medium, CO co-culture group, CT control group. ***p value < 0.001, ns *p* value > 0.05. *n* = 3. **A**–**C** Enhanced endoplasmic reticulum tracker MFI in MDA-MB-231 and BT-549 cells co-cultured with MCF7 cells. **D** Upregulated ERS markers and enhanced apoptosis in MDA-MB-231 and BT-549 cells co-cultured with MCF7 cells and treated with CM. ERS markers and apoptotic markers were suppressed by 4-PBA in MDA-MB-231 and BT-549 cells. **E** Enhanced fluorescence of CHOP detected by immunofluorescence in MDA-MB-231 and BT-549 cells co-cultured with MCF7 cells. **F**–**J** Increased proportions of apoptotic MDA-MB-231 and BT-549 cells co-cultured with MCF7 cells and treated with CM, and no statistical increase in apoptotic proportions was detected in 4-PBA-treated ER− cells. **K** Increased TUNEL-positive stained cells in CM-injected MDA-MB-231 xenografts.

We therefore investigated if ERS was involved in the TAM-induced bystander effect. The enlargement of the endoplasmic reticulum is a typical feature of ERS, which can be visualized by the endoplasmic reticulum tracker [21]. Higher mean fluorescence intensities of a green-fluorescence labeled endoplasmic reticulum tracker were observed in both co-cultured and CM-cultured ER− cells (Fig. 6A–C and Fig. S6A–C), suggesting the functionally active endoplasmic reticulum. Enhanced ERS was further consolidated by western blotting of the ERS marker protein. Upregulated BiP, increased phosphorylation of eukaryotic initiation factor 2α (eIF2α), and elevated expression of CHOP were detected in both co-cultured and CM-cultured ER− cells (Fig. 6D). The phosphorylation of eIF2α has been proved to indicate the activation of a canonical ERS pathway and is the cause of increased CHOP expression [22, 23]. Immunofluorescence assays reinforced the accumulation of CHOP in the nuclei of co-cultured MDA-MB-231 and BT-549 cells (Fig. 6E).

Due to the fact that accumulated CHOP expression triggers cell apoptosis [24–26], we examined whether ERS-induced apoptosis plays a role in bystander tumor suppression. Apoptotic markers, including increased Bax expression and decreased Bcl-2 expression were detected in co-cultured and CM-cultured ER− cells by western blotting. However, no statistical difference of apoptotic markers was detected in ER− cells when ERS was inhibited with the ERS inhibitor 4-Phenylbutyric acid (4-PBA), indicating that apoptosis was dependent on ERS (Fig. 6D). Additionally, no upregulation of GDF15 was detected in the presence of 4-PBA, further proving the upregulated GDF15 resulted from ERS and accorded with the stress-responsive nature of GDF15. In flow cytometry assays, elevated co-cultured and CM-cultured cell apoptosis proportions were accessed by annexin V-FITC/PI staining. Apoptosis of ER− cells was inhibited in the presence of 4-PBA, which is consistent with the results in western blotting (Fig. 6F–J). As for in vivo tests, increased numbers of TUNEL-positive stained MDA-MB-231 cells were witnessed in CM-injected mice subcutaneous tumor tissues (Fig. 6K). Taken together, the attenuation of ER− cells in bystander effect results from activated BiP/eIF2α/CHOP axis and ERS-induced apoptosis.

### PPIB independently promotes ERS-induced apoptosis to kill tumor cells

**Fig. 7:**
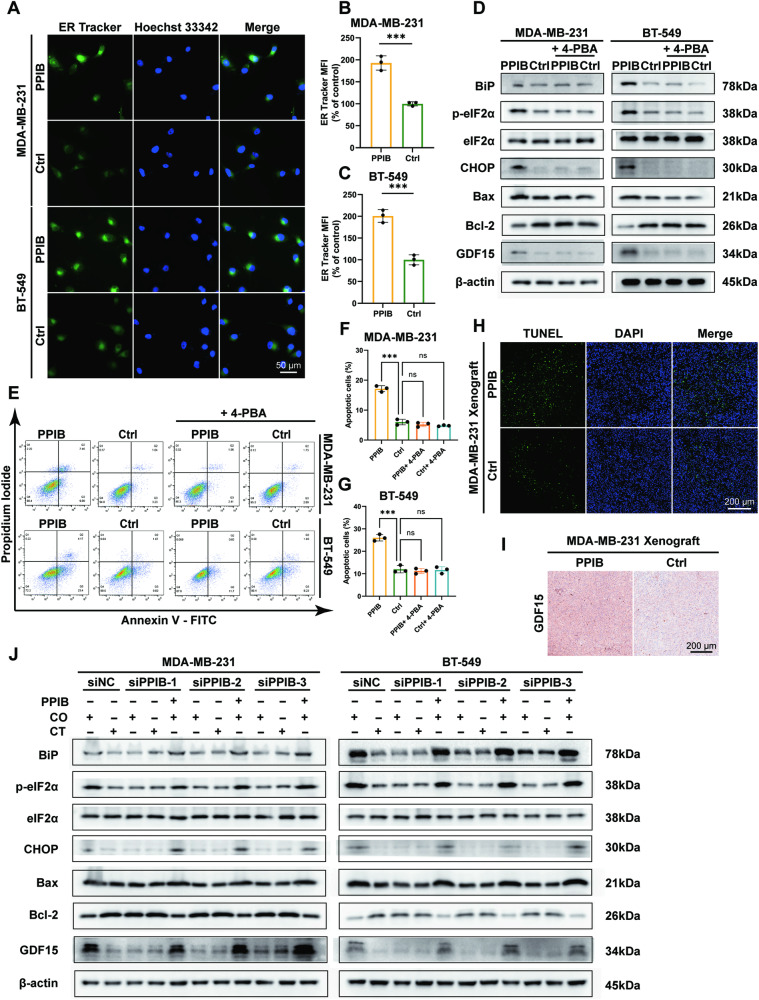
PPIB independently promotes ERS-induced apoptosis to kill tumor cells. CO co-culture group, CT control group. ***p value < 0.001, ns *p* value > 0.05. *n* = 3. **A**–**C** Enhanced endoplasmic reticulum tracker MFI in PPIB-treated MDA-MB-231 and BT-549 cells. **D** Upregulated ERS markers and apoptotic markers in PPIB-treated MDA-MB-231 and BT-549 cells, and ERS-induced apoptosis can be suppressed by ERS inhibitor 4-PBA. **E**–**G** Increased proportions of apoptotic MDA-MB-231 and BT-549 cells treated with PPIB, and no increase in apoptosis in cells treated with 4-PBA. **H** Increased TUNEL-positive stained cells in PPIB-injected MDA-MB-231 xenografts. **I** Enhanced GDF15 expression in PPIB-injected MDA-MB-231 xenografts. **J** Upregulated ERS markers and GDF15 were not detected in MDA-MB-231 and BT-549 cells co-cultured with PPIB-knockdown MCF7 cells while reintroducing PPIB into the medium re-activated TAM-induced bystander effect.

Antitumor protein PPIB has been identified by us to play an antitumor role in bystander effect; thus, we investigated if PPIB can independently kill tumor cells by promoting ERS-induced apoptosis. Treatment with recombinant PPIB protein resulted in increased endoplasmic reticulum tracker fluorescence intensities in MDA-MB-231 and BT-549 cells (Fig. 7A–C). ERS markers were detected by western blotting, and increased expression of BiP, p-eIF2α, and CHOP were found in PPIB-treated MDA-MB-231 and BT-549 cells (Fig. 7D). Consistently, increased CHOP fluorescent intensities were observed in the nuclei of PPIB-treated MDA-MB-231 and BT-549 cells, providing further evidence of the induction of ERS by PPIB (Fig. S7A). The upregulation of apoptosis-related protein Bax and the downregulation of Bcl-2 were also verified, and no aberrant expressions of Bax and Bcl-2 were observed in the presence of 4-PBA. Expressions of the stress-responsive protein GDF15 were explored and correlated with the existence of ERS (Fig. 7D). The flow cytometry assays demonstrated increased apoptotic cell proportions in MDA-MB-231 and BT-549 cells treated with PPIB, whereas inhibited apoptosis was noticed with the addition of 4-PBA (Fig. 7E–G). In PPIB-injected mice subcutaneous tumor tissues, the elevated ratio of TUNEL-positive cells was witnessed, and increased expressions of GDF15 were probed (Fig. 7H, I).

To exclude the impact of complex components in CM and coculturing system on ER− cells, we tested ERS-induced apoptosis in the absence of PPIB in MCF7 CM. Both culturing ER− cells with PPIB-deficient CM and coculturing ER− cells with PPIB-knockdown MCF7 cells triggered no elevated ERS in MDA-MB-231 and BT-549 cells, and reintroducing PPIB into the culture medium rescued these results (Fig. 7J and Fig. S7B). All three sequences of siRNAs knocking down PPIB in MCF7 cells led to consistent results, validating PPIB the primary antitumor component that independently promotes ERS-induced apoptosis in TAM-induced bystander effect.

## Discussion

**Fig. 8:**
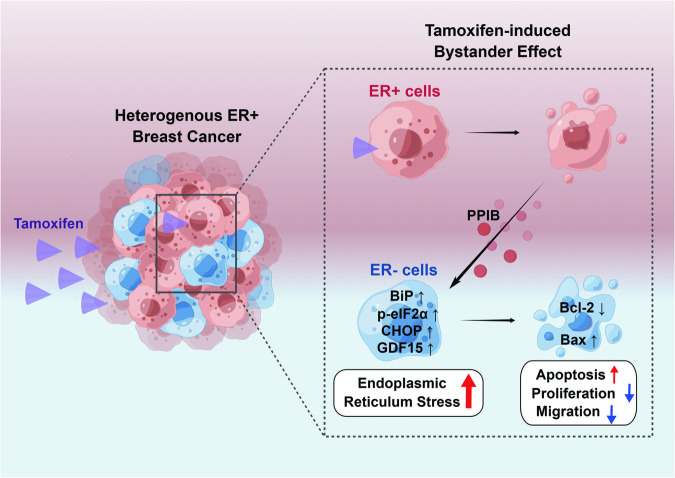
Diagram of the TAM-induced bystander effect in ER+ breast cancer. In response to TAM, ER+ breast cancer cells derive PPIB to selectively attenuate the tumorigenesis of ER− cells. The BiP/eIF2α/CHOP axis in ER− cells is activated, leading to ERS-induced apoptosis.

In this study, we identified a novel bystander effect in TAM treatment, wherein ER+ breast cancer cells selectively attenuated the tumorigenesis of ER− cells. We further revealed that PPIB, the major antitumor protein, enhances the activation of the BiP/eIF2α/CHOP axis, leading to irreversible and overwhelming ERS. Consequently, ERS-induced apoptosis of ER− cells is promoted, while cell viability, proliferation, colony formation, and migration abilities are suppressed (Fig. 8).

In ER+ breast cancer, ER signaling is important in orchestrating the development and progression of breast tumors [27]. Interrupting ER signaling has been one of the most successful therapeutic strategies in cancer therapy, among which TAM has been the most widely administered endocrine therapy for ER+ breast cancer, especially for premenopausal women patients [28]. However, there remains an obvious puzzle in the clinical practice of endocrine therapy for ER+ breast cancers: ER− cells are present with proportions varying from 0 to 99% in tumors defined as ER+ [29, 30]. Little has been known about whether the efficacy of endocrine therapy can override these ER− cells previously, as well as how ER− cells are affected in endocrine therapy. Results from our study shed light on this issue, identifying that ER− cells can be attenuated in the TAM-induced bystander effect. This finding contributes a theoretical foundation for overcoming the innate heterogeneity of ER+ cells by TAM, encouraging TAM monotherapy forward for ER+ breast cancers.

New insights into the bystander effect were also supplied. The bystander effect has been considered an important part of amplifying antitumor therapeutic effects [31, 32]. Previously, it was mainly addressed in radiation therapy and treatment with antibody-drug conjugates. In radiation therapy, irradiated tumor cells can release microparticles that provoke broad antitumor effects [33]. Cathepsin B was reported to promote the cytotoxicity of HER2-targeted antibody-drug conjugates in breast cancer [34]. Our study identified a novel bystander effect in TAM treatment that has not been addressed before, further extending the scope of the bystander effect into endocrine therapy.

As for the mediator of the TAM-induced bystander effect, PPIB was identified as the pivotal antitumor protein. As a brand-new material basis of the bystander effect, PPIB takes part in completing an in-depth understanding of bystander signaling. Extracellularly, PPIB has been proven to be secreted under stress circumstances and can bind with CD147, or upregulate TRAIL receptors [35, 36]. Our results demonstrate that extracellular PPIB also functions as a novel antitumor protein, exhibiting tumor-killing capacity in a dose-dependent manner. Considering the universal expression of PPIB, it is promising to explore PPIB in other tumors and therapies for bystander effects, hence contributing to expanding efficacy.

The attenuating role of the TAM-induced bystander effect was attributed to ERS, which is triggered by accumulated and overloaded misfolded protein in the endoplasmic reticulum lumen [37]. ERS can be either prosurvival or prodeath in the development of breast cancer. External and internal triggers, including hypoxia, undernutrition, and low pH, contribute to aberrant activation of ERS sensors and mild ERS in breast cancer cells. However, ERS is lethal when the accumulation of misfolded protein exceeds the tolerance threshold [38]. In breast cancer treatment, antitumor drugs that induce cell death by activating key modulators in excessive ERS signaling have been explored. Ilamycin E was proven to activate the CHOP/Bcl-2 axis and promote apoptosis of breast cancer cells [39]. AMC-04 was identified as an activator of ATF4/CHOP/DR5 signaling, thereby enhancing therapeutic effects [40]. In our study, the activation of the BiP/eIF2α/CHOP pathway that indicates ERS-induced apoptosis was demonstrated. Increased apoptotic cell proportions and ERS-induced apoptosis independently promoted by PPIB in breast cancer cells were also observed. Results from our study provide a further theoretical basis and potential drug candidate for inducing overwhelmed ERS to treat breast cancer.

Taken together, our study unveiled and deciphered a novel bystander-killing effect on ER− cells in TAM treatment, which can be attributed to the ERS-induced apoptosis mediated by ER+ cells-derived PPIB. The specific nature of this bystander effect on ER− cells was also addressed. Comprehending the TAM-induced bystander effect offers novel insight into overcoming the heterogeneity of ER+ breast cancer. From a therapeutical perspective, the extracellular nature of PPIB suggests a practical and accessible approach to antitumor therapy. Regarding the feasibility of utilizing PPIB as a synergistic antitumor agent or even substitute endocrine therapy in heterogenous ER+ breast cancer, further studies are warranted.

## Materials and methods

### Cell culture

All cell lines were purchased from the American Type Culture Collection (Manassas, VA, USA). MCF10A cells were cultured following the manufacturer’s instructions. MCF7, MDA-MB-231, and HUVEC cells were cultured in Dulbecco’s modified Eagle’s medium (DMEM; Gibco, Carlsbad, CA, USA) with 10% fetal bovine serum (FBS; VivaCell, Shanghai, China). T47D and BT-549 cells were grown in RPMI 1640 medium (Gibco, Carlsbad, CA, USA) with 10% FBS. Cells were identified by STR profiling and tested to exclude mycoplasma contamination. All cells were cultured in a 5% CO_2_ incubator under humidified 37 °C conditions.

### Collection of conditioned medium

About 6 × 10^5^ MCF7 cells were seeded into six-well plates with 1.5 mL medium and exposed to 18 μM 4-hydroxy tamoxifen (TAM; MCE, HY-16950, NJ, USA) or control DMSO. After exposure for 24 h, cells were rinsed with PBS and then maintained in 1 mL complete medium. Another 24 h later, the conditioned medium (CM) was collected and centrifuged to remove any cells or debris. To culture ER− cells, the conditioned medium was mixed with the complete medium at a ratio of 1:1 in case of nutrient exhaustion.

The conditioned medium of T47D was also collected using the above-mentioned approach.

### Co-culture systems

Co-culture of ER− cells and MCF7 cells was conducted using transwell cell culture chambers (Corning Inc., Tewksbury, MA, USA) with a polycarbonate membrane insert (0.4 µm pore size). ER− cells were plated in the lower chamber and MCF7 cells were plated in the insert. Cells were exposed to 18 μM TAM 24 h post-plating. Co-culture systems would be maintained for 72 h after TAM exposure, and ER− cells were harvested.

### Cell proliferation assays

Cell Counting Kit-8 (CCK-8) assays and 5-ethynyl-2’-deoxyuridine (EdU) assays were conducted to assess the cell viability and proliferation ability. CCK-8 assays were performed under the manufacturer’s instructions (Bimake, B34302, Houston, TX, USA). The EdU assay kit was purchased from Ribobio (Guangzhou, China). EdU solution, Apollo 567 and 4’,6-diamidino-2-phenylindole (DAPI; Servicebio, Wuhan, China) were used to stain cell nuclei subsequently. For visualization, cells were imaged by a fluorescence microscope at the wavelengths of 594 and 340 nm (Carl Zeiss, AXIO observer 7, Germany).

### Wound healing and transwell migration assays

The migration ability of cells was tested by wound healing and transwell assays. For wound healing assays, cells were cultured in six-well plates until the confluence reached 90%. A 200 μL pipette tip was used to scratch the cell. Afterward, scratches were observed and migration areas were measured with a microscope (Olympus, Tokyo, Japan) at 0 and 24 h.

For transwell assays, cells were placed in the upper chamber with serum-free medium (pore size, 8 μm; Corning Inc.), and medium containing FBS was added to the lower chamber. Cells that migrated into the lower chamber were fixed and stained with crystal violet after 24 h. Random fields were imaged by a microscope (Olympus, Tokyo, Japan) and the average number of cells was measured.

### Colony formation assays

To evaluate colony formation ability, 1 × 10^3^ cells were seeded in six-well plates. Cells were allowed to adhere and grow into colonies for about 14 days. Colonies were fixed and stained with crystal violet to be counted.

### Apoptosis analysis

TAM-treated, co-cultured, or PPIB-treated ER− cells were digested with EDTA-free trypsin (Biosharp) and washed with PBS. For detection, cells were resuspended with the binding buffer. The Annexin V-FITC/Propidium Iodide (PI) Apoptosis Detection Kit (BD Biosciences, Franklin Lakes, NJ, USA) was utilized to label cells under the condition of protein from light. Samples were incubated at 4 °C for 30 min and detected with a flow cytometer (LSRFortessa X-20; BD). Data were analyzed with Flowjo software V10 (Flowjo, LLC).

### RNA extraction and real-time quantitative PCR (qPCR)

Total RNA was extracted using standard methods with Trizol (Vazyme, Nanjing, China). After RNA was reverse transcripted to cDNA with HiScript III qRT SuperMix (Vazyme), real-time qPCR was conducted by a BioRad CFX96 Real-Time PCR Detection System (Carlsbad CA, USA). Expression fold changes of mRNA were analyzed subsequently, taking β-actin as an internal control. Primer sequences are listed in Table S1.

### Western blotting

For protein extraction, cells were lysed with RIPA buffer (Biosharp, Hefei, China) containing protease inhibitors (MCE). Proteins were quantified with a BCA assay kit (Vazyme) and western blotting was done using standard procedures, as previously reported [41]. Polyvinylidene fluoride (PVDF) membranes (Millipore, Darmstadt, Germany) were utilized to transfer proteins. We detected signals and acquired images using Electrochemiluminescence (ECL) luminescent solution (Biosharp) and ChemiDoc XRS+ imaging system (BioRad). Antibodies against PCNA (Servicebio, GB11010), E-cadherin (Abclonal, A20798; Wuhan; China), N-cadherin (Abclonal, A19083), BiP (Cell Signaling Technology, 3177 S; Danvers; MA; USA), eIF2α (Cell Signaling Technology, 2103 S), p-eIF2α (Abclonal, AP0692), CHOP (Proteintech, 15204-1-AP; Wuhan; China), GDF15 (Abclonal, A0185), PPIB (Abcam, ab178397; Cambridge; MA; USA), Bax (Abclonal, A0207), Bcl-2 (Abclonal, A0208), β-actin (Cell Signaling Technology, 8457 S) were used in our study. Original Western blots are also shown as supplementary information.

### Label-free quantitative proteomics

Label-free Quantitative Proteomics was conducted at Bioprofile Technology (Shanghai, China). Serum-free supernatant of TAM-treated or DMSO-treated MCF7 cells was collected 24 h after drug elution (*n* = 3 for each group). Samples were lyophilized, lysed, and boiled, followed by 2 min of ultrasonication. Lysates were centrifuged at 16,000×*g* for 20 min and redissolved with 200 µL UA (8 M Urea, 150 mM Tris-HCl). In addition to trypsin digestion, extracting peptides, and column separation, a Q-Exactive HF-X mass spectrometer (Thermo Scientific) was used to analyze the peptides. Using Proteome Discoverer 2.4 (Thermo Scientific), protein sequences were identified according to the UniProt database Homo sapiens (Human) [9606]-20598-20220803. fasta. At least one unique peptide was present in each identified protein. The Peptide Spectrum Matches (PSMs) false discovery rate (FDR) and protein FDR were both set as <0.01. Statistically differentially expressed proteins were identified with a filter of *p* value <0.05, |log2FC| >2. The mass spectrometry proteomics data of our study have been deposited to the ProteomeXchange Consortium (http://proteomecentral.proteomexchange.org) via the iProX partner repository with the dataset identifier PXD047000.

### ELISA and PPIB recombinant protein treatment

The concentration of PPIB in cell supernatant was tested with an ELISA kit purchased from JiangLai Biological (JL11615, Shanghai, China). ELISA assays were conducted following the manufacturer’s instructions.

PPIB recombinant protein (Proteintech, Ag17957) was resolved and diluted with sterile water. Cells were treated with PPIB recombinant protein at concentrations of 125, 250, and 500 ng/mL.

### RNA sequencing

As sequencing libraries, Illumina NEBNext Ultra^TM^ RNA Library Prep Kit for Illumina® (NEB, USA) was used. The sequencing of RNA was carried out by Novogene (Beijing, China) following standard procedure. Different expressions of genes were considered to be statistically significant if *p* <0.05. Data files for RNA sequencing were uploaded to the NCBI Gene Expression Omnibus databases (GSE247920).

### Small interfering RNA (siRNA) transfection assays

For siRNA transfection, lipofectamine 3000 (Invitrogen) and OptiMEM (Gibco) were used, following the manufacturer’s instructions. Control siRNA (siNC) and siPPIB were synthesized by Qingke Biotechnology (Wuhan, China). The sequence of siNC is UUCUCCGAACGUGUCACGUTT. The sequences of siPPIB were CCUACGAAUUGGAGAUGAA (siPPIB-1), CAGCAAUUCCAUCGUGUA (siPPIB-2), and GCCUUAGCUACAGGAGAGA (siPPIB-3).

### Fluorescent labeling with endoplasmic reticulum tracker green

To label the endoplasmic reticulum, cells were incubated with 1 µM endoplasmic reticulum tracker green (Beyotime, C1042S, Shanghai, China) for 30 min according to the manufacturer’s instructions. Cells were then fixed, and nuclei were stained with Hoechst 33342 (Ribobio). The fluorescence was visualized by an AXIO observer 7 microscope (Carl Zeiss).

### Immunofluorescence

Immunofluorescence assays were done to detect the location and expression of proteins. Cells were planted in confocal dishes and fixed with paraformaldehyde after adhesion. A cy3-conjugated goat anti-rabbit IgG (H + L) antibody (Proteintech) was used as a secondary antibody, and the nucleus was dyed with DAPI (Servicebio). Fluorescence was visualized by an AXIO observer 7 fluorescence microscope (Carl Zeiss).

### Animal experiments

Animal experiments were executed under the permission and supervision of the Animal Care Committee of Tongji Medical College ([2022] IACUC Number: 3359). Female BALB/c nude mice at the age of 4 weeks old were purchased from Vital River Laboratory Animal Technology Co. Ltd. (Beijing, China). MDA-MB-231 cells (2 × 10^6^) mixed with matrix gel (BD) at a ratio of 1:1 were injected into bilateral axillary flanks of nude mice to develop subcutaneous tumor xenograft models. When tumors reached 100–150 mm^3^, nude mice were randomized to intratumorally inject fresh-collected, serum-free CM or CN, or PPIB or PBS 100 µL once per day for 7 days (*n* = 8 per group) [42–44]. To assess tumor growth, the longest diameters (D) and shortest diameters (d) of tumors were measured every 3 days with a caliper. Tumor volumes were calculated by the formula (d^2^ × D)/2 [45]. Mice were sacrificed once tumor volume reached 1000 mm^3^, and tumors were dissected to be weighed and undergo subsequent experiments.

### IHC and TUNEL staining

Paraformaldehyde-fixed tissues were embedded in paraffin. Sections were cut at a width of 3–5 µm and dewaxed.

For IHC assays, Citrate buffer was used to unmask antigens, while 3% hydrogen peroxide was used to deplete endogenous peroxidases. Antibodies against Ki-67 (Servicebio, GB111499), PCNA (Servicebio, GB11010), and GDF15 (Abclonal, A0185) were used according to standard techniques. Slides were randomly imaged by a microscope (Olympus).

For TUNEL staining, sections were permeabilized with protease K. Apoptotic cells were detected with a TUNEL BrightGreen Apoptosis Detection Kit (Vazyme, A112) under the guidelines of the manufacturer.

### Statistical analysis

Data were presented as mean ± standard deviation (SD) or as values. The significance of the difference was evaluated by grouped two-tailed Student’s *t*-tests. Pictures were measured and analyzed with ImageJ software. Statistical analyses were conducted using SPSS 24.0 (SPSS Inc. Chicago, IL, USA) and GraphPad Prism 9.0 software (GraphPad Software, San Diego, CA). It was considered statistically significant if the *p* value was less than 0.05 (*), 0.01 (**), or 0.001 (***).

### Reporting summary

Further information on research design is available in the Nature Research Reporting Summary linked to this article.

### Supplementary information

Supplementary Figures and Table

Reporting Summary

## Data Availability

All data are available in the article and supplementary information. The mass spectrometry proteomics data of our study have been deposited to the ProteomeXchange Consortium (http://proteomecentral.proteomexchange.org) via the iProX partner repository with the dataset identifier PXD047000. Data files for RNA sequencing were uploaded to the NCBI Gene Expression Omnibus databases (GSE247920) and will be public from the date of publication. Original Western blots are also shown as supplementary information.
