# Supplementary material for: A novel bystander effect in tamoxifen treatment: PPIB derived from ER+ cells attenuates ER− cells via endoplasmic reticulum stress-induced apoptosis
Source: Cell Death Dis. 2024 Feb 15;15(2):147. doi: 10.1038/s41419-024-06539-3 (PMC10869711; doi:10.1038/s41419-024-06539-3)
Supplement: Supplementary file 1 — Supplementary Figures and Table [file 41419_2024_6539_MOESM1_ESM.pdf]

Supplementary Material

A novel bystander effect in tamoxifen treatment: PPIB derived from ER+ cells attenuates ER- cells via endoplasmic reticulum stress-induced apoptosis

Tinglin Yang[1, #], Wenhui Li[1, #], Jun Zhou[1, #], Ming Xu[1], Ziwei Huang[1], Jie Ming[1, *], Tao Huang[1, *]

This file includes:

Figure S1-S7

Table S1

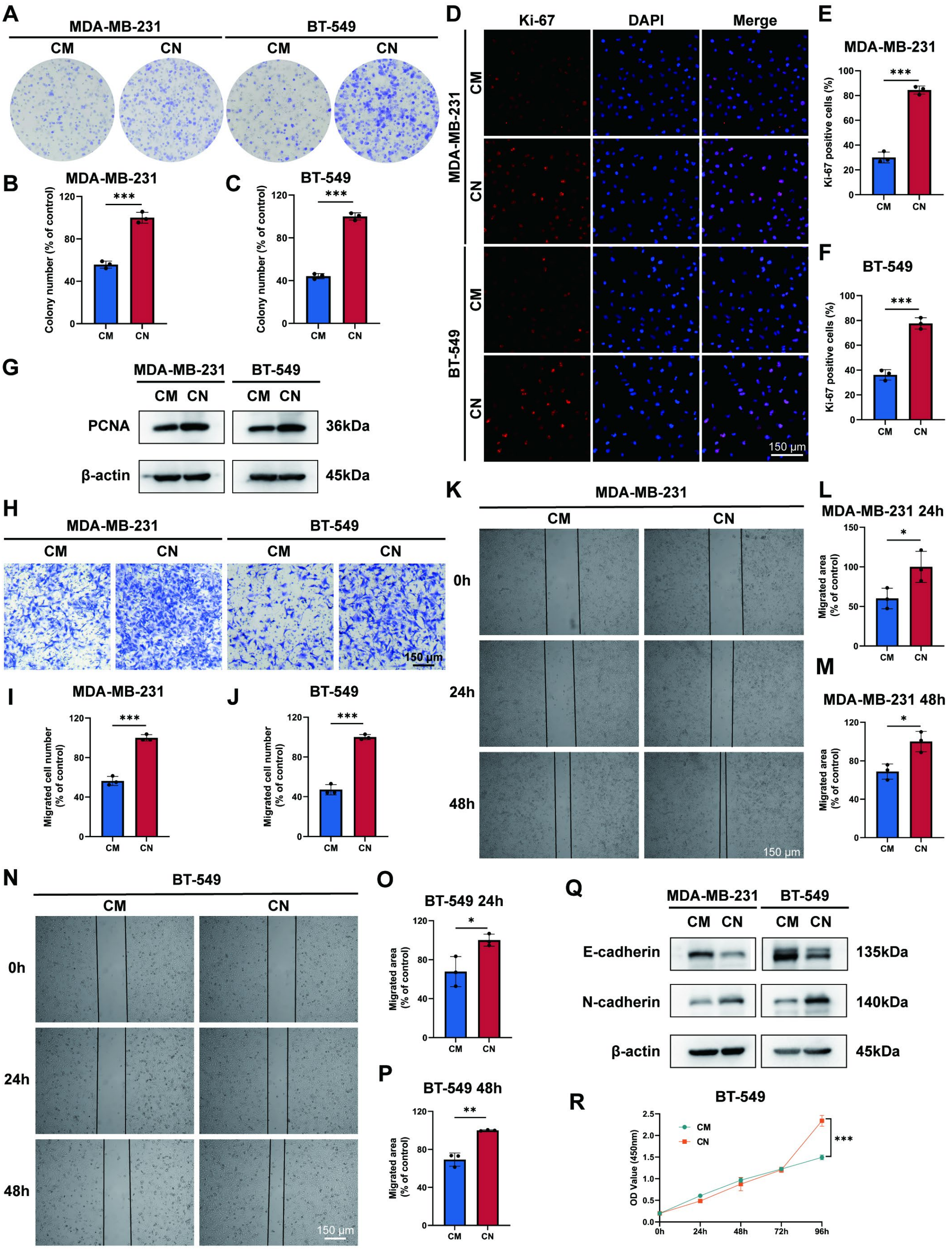

Figure S1. Conditioned medium from TAM-treated ER+ cells attenuates tumorigenesis of ER- cells. CM: conditioned medium; CN: ctrl medium. *p-value < 0.05, **p-value < 0.01, ***p-value < 0.001.

(A-C) ER- breast cancer cell lines MDA-MB-231 and BT-549 were cultured with CM or CN

generated from MCF7 cells. The colony-forming capacity was repressed in CM-cultured MDA-MB-231 and BT-549 cells. n=3. **(D-F)** Decreased percentage of Ki-67 positive cells were detected in CM-cultured MDA-MB-231 and BT-549 cells. n=3. **(G)** PCNA expressions were reduced in CM-cultured MDA-MB-231 and BT-549 cells. n=3. **(H-J)** Transwell-based migration was inhibited in CM-cultured MDA-MB-231 and BT-549 cells. n=3. **(K-M)** Reduced migrated areas at both 24 hours and 48 hours were detected in CM-cultured MDA-MB-231 cells in wound healing assays. n=3. **(N-P)** Reduced migrated areas at both 24 hours and 48 hours were detected in CM-cultured BT-549 cells in wound healing assays. n=3. **(Q)** Increased E-cadherin levels and decreased N-cadherin levels were detected in CM-cultured MDA-MB-231 and BT-549 cells. n=3. **(R)** Attenuated viability was measured in BT-549 cells cultured with CM generated from T47D cells. n=5.

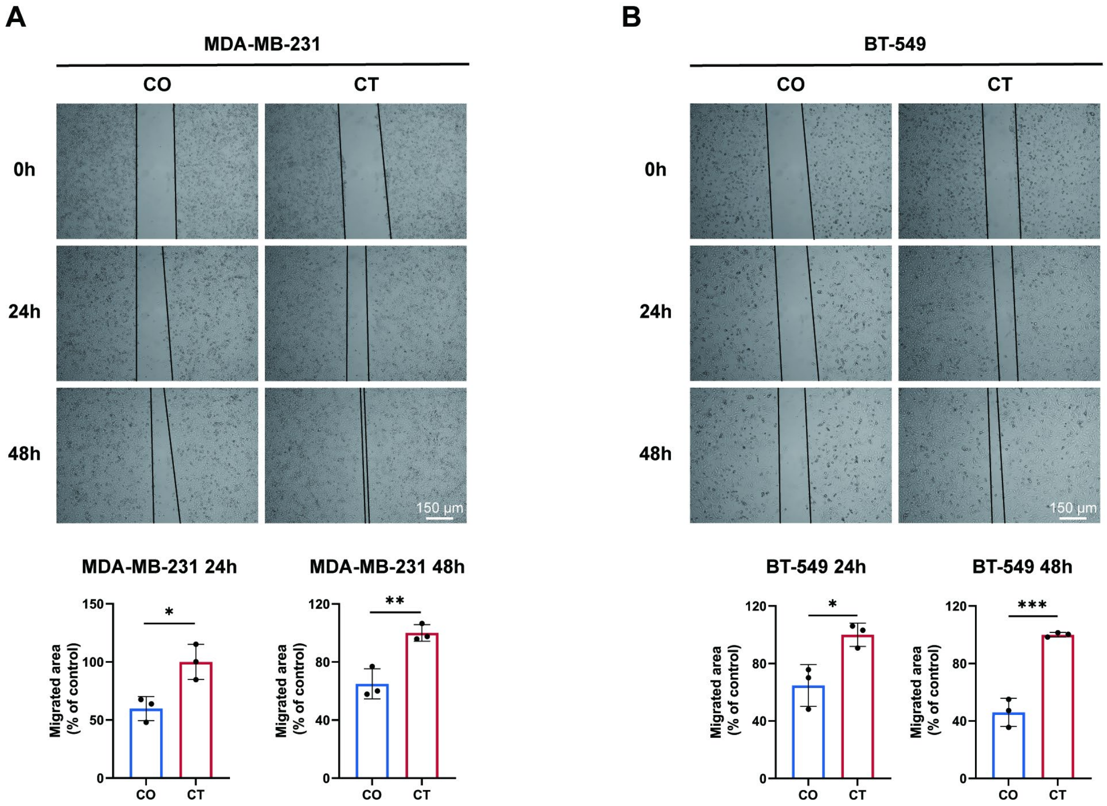

Figure S2. Attenuated migration ability of ER- cells co-cultured with MCF7 cells. CO: co-culture group; CT: control group. *p-value < 0.05, **p-value < 0.01, ***p-value < 0.001. n=3. **(A)** Reduced migrated areas at both 24 hours and 48 hours were detected in MDA-MB-231 cells co-cultured with MCF7 cells. **(B)** Reduced migrated areas at both 24 hours and 48 hours were detected in BT-549 cells co-cultured with MCF7 cells.

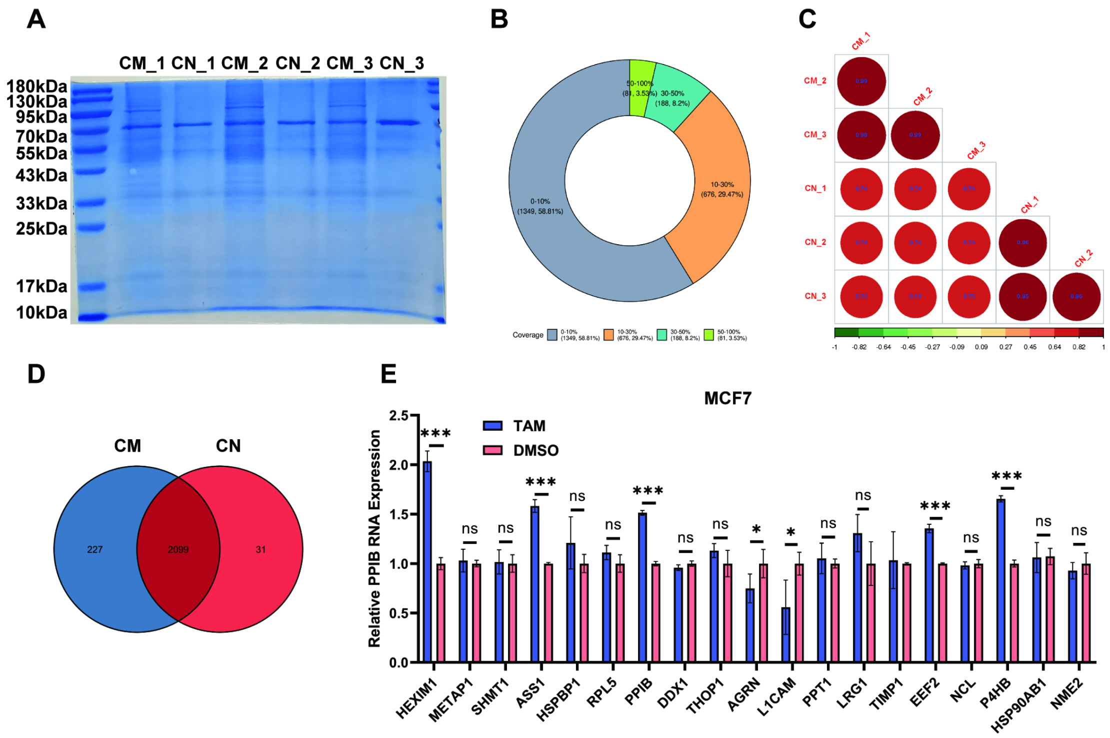

Figure S3. Label-free quantitative proteomics of MCF7 CM and CN. CM: conditioned medium; CN: control medium. *p-value < 0.05, ***p-value < 0.001. n=3.

(A) MCF7 cells were treated with TAM or DMSO to generate serum-free CM or CN. More protein bands and greater protein concentrations are stained in CM groups. **(B)** Peptide coverage of proteins in mass spectrometry. **(C)** Bubble plot exhibiting correlations of samples. **(D)** Venn diagram demonstrating common and unique proteins. **(E)** Validation of top candidates by qPCR.

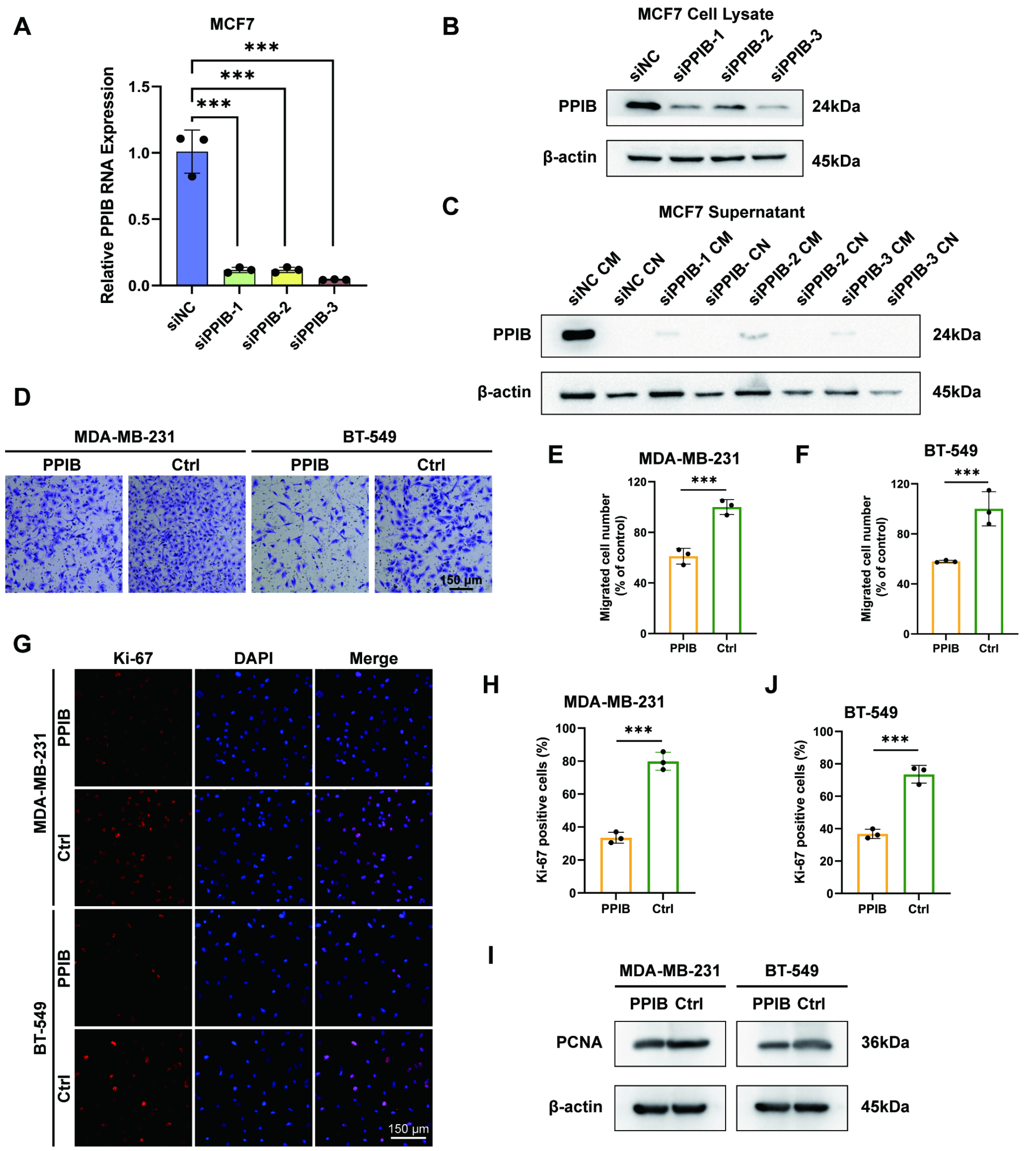

Figure S4. PPIB is the predominant antitumor protein in MCF7 CM. CM: conditioned medium; CN: control medium. ***p-value < 0.001. n=3.

(A-B) Successful knockdown of PPIB in MCF7 cells validated by qPCR and western blotting. **(C)** Decreased PPIB levels in CM by knocking down PPIB in MCF7 cells. **(D-F)** Impaired transwell-based migration in PPIB-treated MDA-MB-231 and BT-549 cells. **(G-J)** Reduced percentage of Ki-67 positive cells was detected in PPIB-treated MDA-MB-231 and BT-549 cells. **(I)** PCNA expressions in MDA-MB-231 and BT-549 cells were suppressed by PPIB treatment.

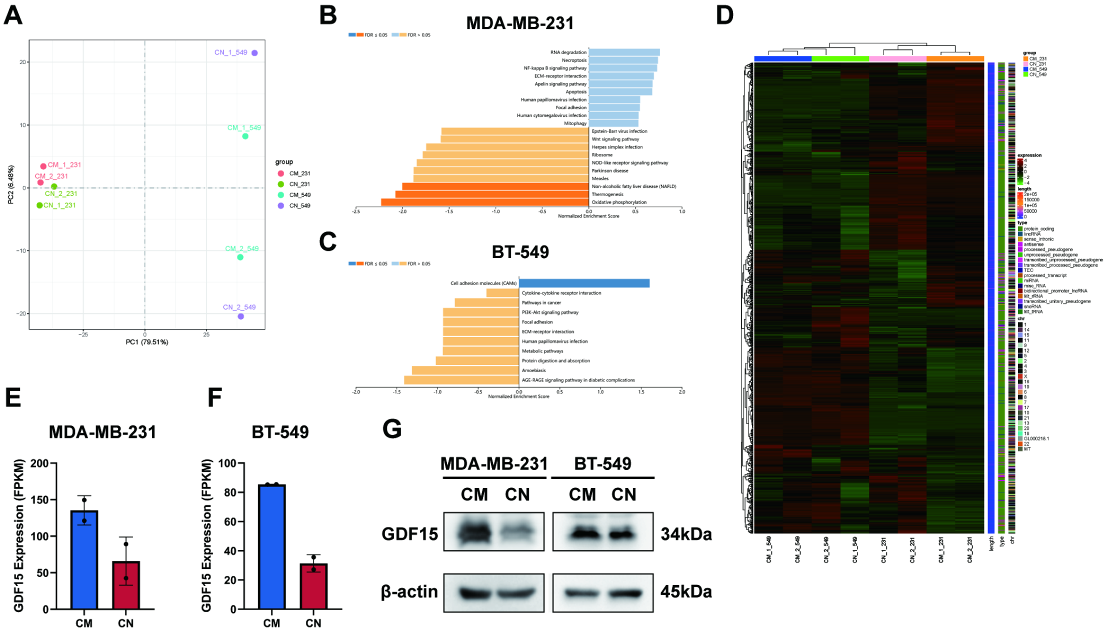

Figure S5. Stress-responsive protein GDF15 is upregulated in ER- cells in TAM-induced bystander effect. CM: conditioned medium; CN: control medium.

(A) PCA analysis of MDA-MB-231 and BT-549 cells cultured with CM or CN. **(B-C)** Top enriched KEGG pathways in MDA-MB-231 and BT-549 cultured with CM. **(D)** Heat map displaying cluster analysis of DEGs within and between groups. **(E-F)** GDF15 RNA levels in RNA sequencing (FPKM). **(G)** Increased GDF15 expressions in MDA-MB-231 and BT-549 cells cultured with CM.

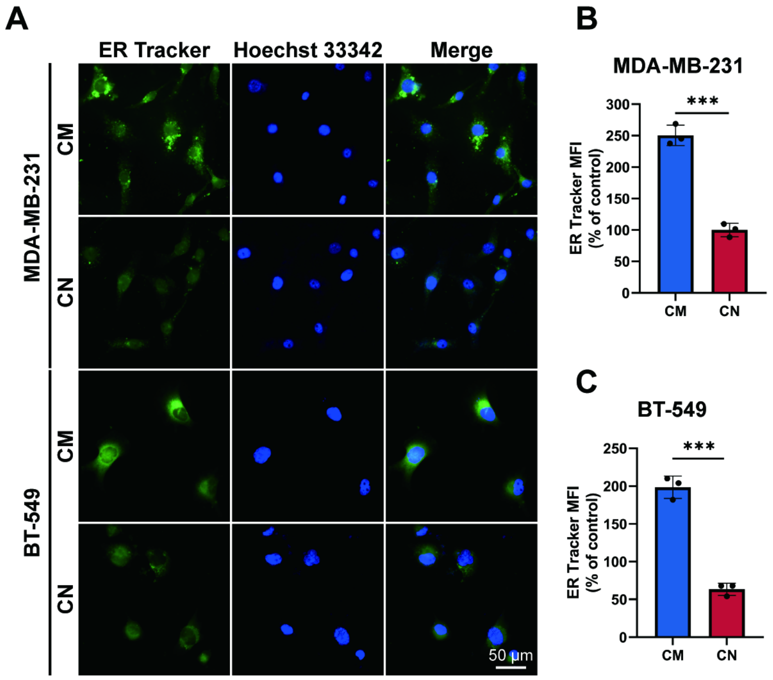

Figure S6. ERS-induced apoptosis is activated in ER- cells cultured with CM. CM: conditioned medium; CN: control medium. ***p-value < 0.001. n=3.

(A-C) Enhanced endoplasmic reticulum tracker MFI in CM-cultured MDA-MB-231 and BT-549 cells.

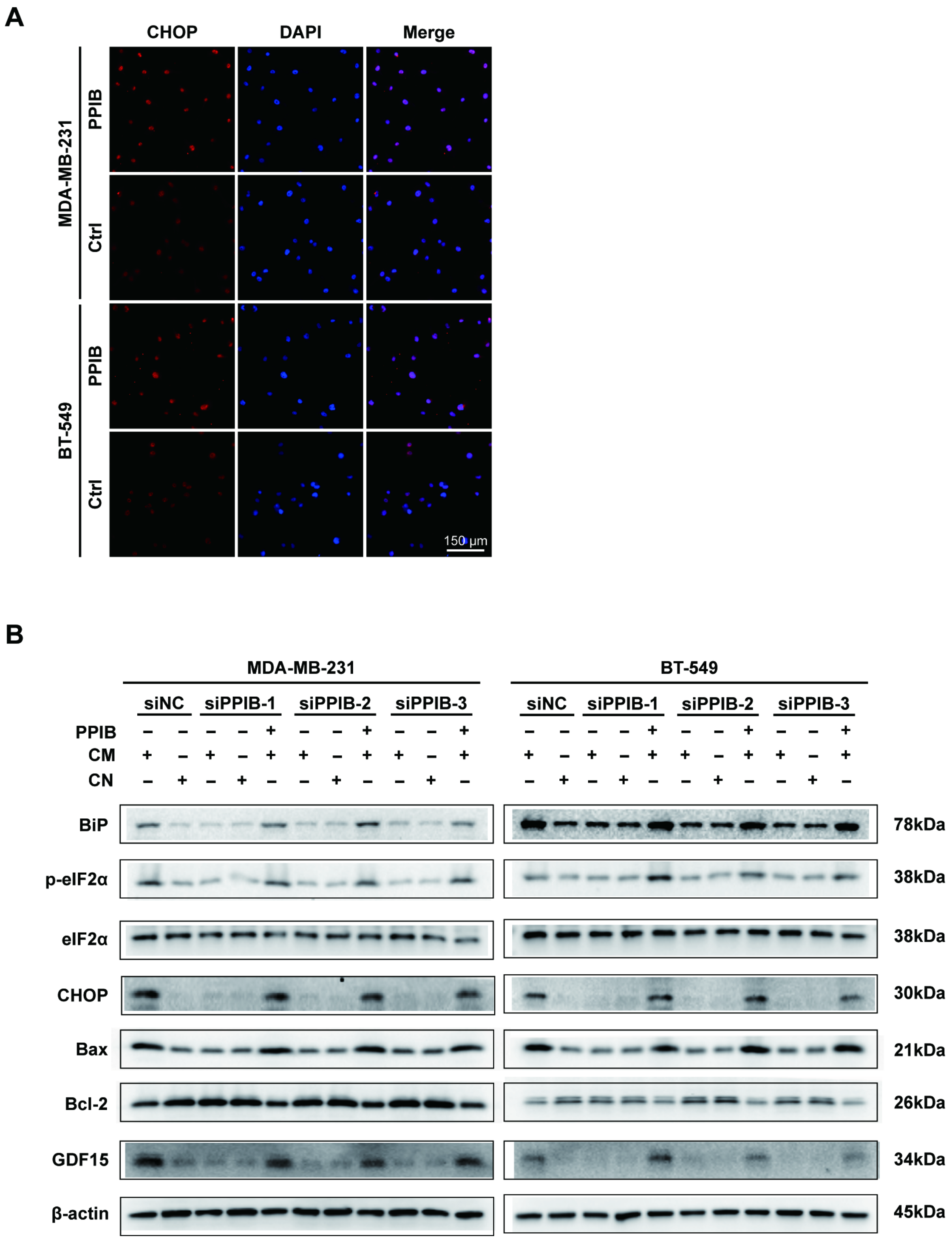

Figure S7. PPIB independently promotes ERS-induced apoptosis to kill tumor cells. CM: conditioned medium; CN: control medium. n=3.

(A) Increased fluorescence of CHOP detected by IF in PPIB-treated MDA-MB-231 and BT-549 cells. **(B)** Upregulated ERS markers and GDF15 were not detected in MDA-MB-231 and BT-549 cells cultured with PPIB-deficient MCF7 CM, while reintroducing PPIB into CM re-activated TAM-

induced bystander effect.

Table S1. Sequences of primers used in the study.

Gene	Forward Primer	Reverse Primer
HEXIM1	CCGAGGCCAGTAAGTTGGG	GACGGGCGTCTCCTATGTTT
METAP1	AAGGGATGCGACTTGTATGTAGG	CTTCTTGTAAGGGCCTTCTGTC
SHMT1	CTGGCACAACCCCTCAAAGA	AGGCAATCAGCTCCAATCCAA
ASS1	CTTGGGGCCAAAAAGGTGTTC	GAGGTAGCGGTCCTCATACAG
HSPBP1	CAGAACCGATGAGTGAGGAGA	TCTGCGGCATTGTCCATGTT
RPL5	GCTCGGAAACGCTTGGTGATA	CCCTCTATACGGGCATAAGCAAT
PPIB	AAGTCACCGTCAAGGTGTATTTT	TGCTGTTTTTGTAGCCAAATCCT
DDX1	TCTCCGAGATGGGTGTAATGC	ACCTCCTCCTAAGATCAATGGG
THOP1	CAAGCTGGGCCGGAGAAAT	TGTTGAAGTCGATGCACAGAAG
AGRN	ACGGAGTCACCTACGAAAACG	AGCAATCACTGTCATACGTGC
L1CAM	TGTCATCACGGAACAGTCTCC	CTGGCAAAGCAGCGGTAGAT
PPT1	GGAGCGGGGTATCAATGAGTC	CCACTCCGAATCTACAGGGTC
LRG1	GGACACCCTGGTATTGAAAGAAA	TAGCCGTTCTAATTGCAGCGG
TIMP1	CTTCTGCAATTCCGACCTCGT	ACGCTGGTATAAGGTGGTCTG
EEF2	CATTGCCGAGCGCATCAAG	CTCGCCGTAGGTGGAGATGA
NCL	GCACCTGGAAAACGAAAGAAGG	GAAAGCCGTAGTCGGTTCTGT
P4HB	GGCTATCCCACCATCAAGTTC	TCACGATGTCATCAGCCTCTC
HSP90AB1	CATCTCCATGATTGGGCAGTT	CTTTGACCCGCCTCTCTTCTA
NME2	AGACCGACCATTCTTCCCTG	TGATGTTCCTGCCAACCTGA
IFI6	GGTCTGCGATCCTGAATGGG	TCACTATCGAGATACTTGTGGGT
IFI44	TTTTCGATGCGAAGATTCACTGG	CCTGATGCGTTACATGCCCTT
ABCA1	ACCCACCCTATGAACAACATGA	GAGTCGGGTAACGGAAACAGG
IGFBP4	GAGCTGGGTGACACTGCTTG	CCCACGAGGACCTCTACATCA
IGFBP7	CGAGCAAGGTCCTTCCATAGT	GGTGTCGGGATTCCGATGAC
SREBF1	CGGAACCATCTTGGCAACAGT	CGCTTCTCAATGGCGTTGT
PRSS23	CAGTGTCATAAGGGAACTCCAC	CCTGAGTCTCGGTGTTGGG
GDF15	ACCTGCACCTGCGTATCTCT	CGGACGAAGATTCTGCCAG
β-actin	TGGCACCCAGCACAATGAA	CTAAGTCATAGTCCGCCTAGAAGCA
